# Piezo1-mediated stellate cell activation causes pressure-induced pancreatic fibrosis in mice

**DOI:** 10.1172/jci.insight.158288

**Published:** 2022-04-22

**Authors:** Sandip M. Swain, Joelle M-J Romac, Steven R. Vigna, Rodger A. Liddle

**Affiliations:** 1 Department of Medicine, Duke University, Durham, North Carolina, USA.; 2 Department of Veterans Affairs Healthcare System, Durham, North Carolina, USA.

**Keywords:** Gastroenterology, Fibrosis

## Abstract

Pancreatic fibrosis is a complication of chronic pancreatitis and is a prominent feature of pancreatic cancer. Pancreatic fibrosis is commonly observed in patients with prolonged pancreatic duct obstruction, which elevates intrapancreatic pressure. We show here that increased pancreatic duct pressure causes fibrosis and describes the mechanism by which pressure increases deposition of extracellular matrix proteins and fibrosis. We found that pancreatic stellate cells (PSCs), the source of the extracellular matrix proteins in fibrosis, express the mechanically activated ion channel Piezo1. By increasing intracellular calcium, mechanical stress or the Piezo1 agonist Yoda1-activated PSCs manifest by loss of perinuclear fat droplets and increased TGF-β1, fibronectin, and type I collagen expression. These effects were blocked by the Piezo1 inhibitor GsMTx4 and absent in PSCs from mice with conditional genetic deletion of Piezo1 in stellate cells, as was pancreatic duct ligation–induced fibrosis. Although TRPV4 has been proposed to have direct mechanosensing properties, we discovered that PSCs from Trpv4-KO mice were protected against Yoda1-triggered activation. Moreover, mice devoid of TRPV4 were protected from pancreatic duct ligation–induced fibrosis. Thus, high pressure within the pancreas stimulates Piezo1 channel opening, and subsequent activation of TRPV4 leads to stellate cell activation and pressure-induced chronic pancreatitis and fibrosis.

## Graphical abstract

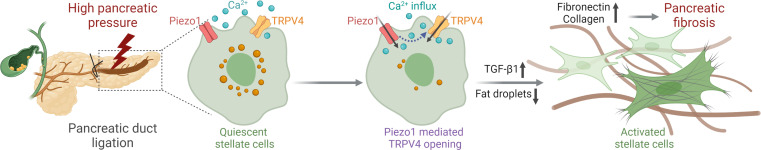

## Introduction

Injury to the pancreas most commonly causes acute pancreatitis, which is accompanied by acinar cell necrosis (1–3); however, recurrent or sustained injury leads to chronic pancreatitis, which may be complicated by exocrine and endocrine insufficiency (4–7). Chronic pancreatitis is a progressive inflammatory disease of the pancreas that is characterized by fibrosis and loss of insulin-producing β cells (6, 8). Quality of life in patients with chronic pancreatitis is reduced due to abdominal pain, pancreatic exocrine insufficiency, and diabetes mellitus (7). Once pancreatic fibrosis develops, recovery of pancreatic function is limited (7). Several studies reported that people with chronic pancreatitis have reduced life expectancy (9), which may be due in part to increased risk of pancreatic cancer — a leading cause of cancer death (9, 10).

Several factors contribute to the development and progression of chronic pancreatitis. Although alcohol consumption is the leading cause of chronic pancreatitis, genetic mutations and pancreatic duct obstruction are other well-recognized contributors (5, 7, 8). Each of these causes results in pancreatic fibrosis, which can lead to scarring and narrowing of large and small pancreatic ducts, both of which lead to increased intrapancreatic pressure. Thus, it appears that elevated duct pressure can cause pancreatic fibrosis and that pancreatic fibrosis can further exacerbate pancreatic pressure.

We recently demonstrated that pancreatic acinar cells sense pressure through the mechanically activated ion channel Piezo1 (11, 12). Piezo1 is a large membrane–spanning protein that resides in many pressure-sensitive cells (13). Membrane tension caused by shear stress, membrane stretching, or high pressure opens the Piezo1 channel, allowing the influx of cations, mainly Ca^2+^ (14–16). Excessive Piezo1 activation produces abnormally high concentrations of intracellular calcium ([Ca^2+^]_i_), which have deleterious effects on cells (11, 13).

Pancreatic fibrosis results from the deposition of extracellular matrix (ECM) proteins that are secreted by activated pancreatic stellate cells (PSCs; refs. 17–20). However, the mechanism by which force initiates these effects is unknown. Based on the observations that duct obstruction leads to pancreatic fibrosis, we postulated that PSCs are also pressure sensitive. We report here that PSCs express the mechanically sensitive ion channel Piezo1, which is responsible for pressure-induced pancreatic fibrosis.

## Results

### Increased intrapancreatic pressure causes pancreatic fibrosis.

**Figure 1:**
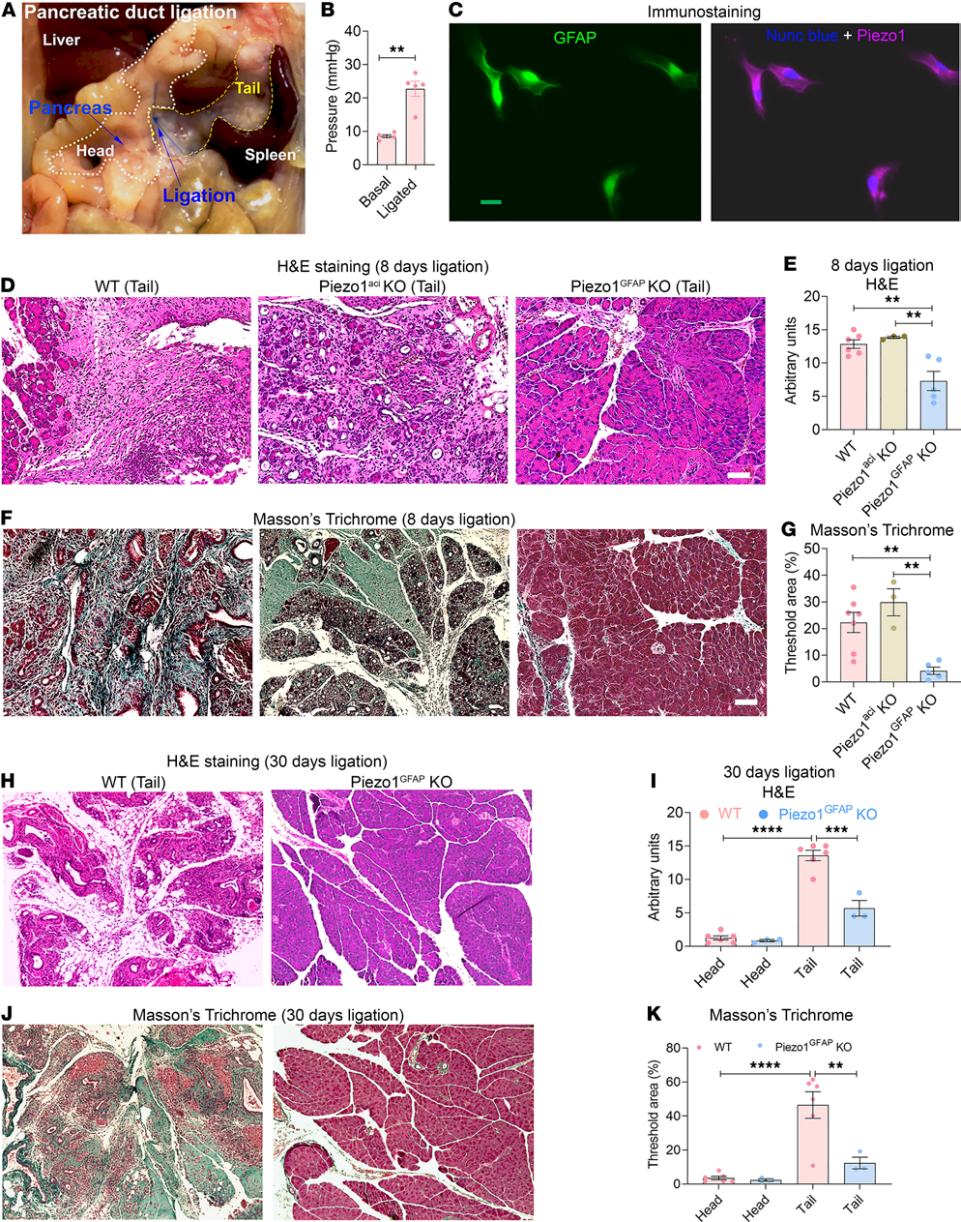
Piezo1^GFAP^-KO mice were protected from pancreatic duct ligation–induced fibrosis. (**A**) Photograph of pancreatic duct ligation (PDL) at the tail region of the pancreas. The head and tail regions of the pancreas are outlined in white and yellow, respectively. (**B**) Pressure in the tail region of the pancreas before and 5 minutes after pancreatic duct ligation. (**C**) Immunostaining of GFAP and Piezo1 in mouse PSCs. (**D**–**G**) Eight days after PDL, chronic pancreatitis and fibrosis parameters included (**D**) tail region H&E staining, (**E**) tail region H&E score, (**F**) tail region Masson’s trichrome staining, and (**G**) tail Masson’s trichrome area of WT, Piezo1^aci^-KO, and Piezo1^GFAP^-KO mice (*n =* 3–7). (**H**–**K**) Thirty days after PDL, chronic pancreatitis and fibrosis parameters included tail region (**H**) H&E staining, (**I**) H&E score, (**J**) tail region Masson’s trichrome staining, and (**K**) Masson’s trichrome area of WT and Piezo1^GFAP^-KO mice (*n =* 3–6). Statistical analyses were calculated using 2-tailed Student’s *t* test for 2 groups, and multiple groups were analyzed by 1-way ANOVA. **P ≤ 0.01, ***P ≤ 0.001; ****P ≤ 0.0001. Scale bar: 100 μm.

We developed a clinically relevant obstructive pancreatitis model by ligating the pancreatic tail region (Figure 1A) to evaluate the effects of intrapancreatic pressure on fibrosis (21). Five minutes after ligation, pancreatic pressure in the tail region increased from 8.5 mmHg (unligated pressure) to 22.7 mmHg (ligated pressure) (Figure 1B). Eight days after pancreatic duct ligation (PDL), substantial pancreatic fibrosis was observed in the tail region of the pancreata. The nonligated head region of the pancreas was unaffected (Figure 1, D–G and J, and Supplemental Figure 1; supplemental material available online with this article; https://doi.org/10.1172/jci.insight.158288DS1). Similar results were observed 30 days after duct ligation (Figure 1, H–K). Accompanying the high deposition of collagen was loss of endocrine and exocrine tissue in the tail region (Figure 1, J and K).

### Piezo1^GFAP^-KO mice were protected from pressure-induced pancreatic fibrosis.

The demonstration that duct obstruction induced pancreatic fibrosis raised the possibility that PSCs were pressure sensitive. Mechanically activated ion channels are key pressure sensors in many tissues, and we previously demonstrated that the most highly expressed mechanoreceptor in the pancreas is Piezo1 (11). To determine possible mechanoreceptor expression, Piezo1 was identified in PSCs by immunostaining (Figure 1C and Supplemental Figure 2, A and B). To evaluate the potential role of Piezo1 in PSCs and pancreatic fibrosis, we deleted *Piezo1* in cells expressing the stellate cell gene glial fibrillary acidic protein (GFAP). Piezo1^GFAP^-KO mice were protected from partial duct ligation–induced pancreatic fibrosis 8 days after ligation (Figure 1, D–G). In contrast, mice with selective deletion of Piezo1 in pancreatic acinar cells (Piezo1^aci^-KO mice) were not protected against pressure-induced fibrosis (Figure 1, D–G). Similarly, 30 days after duct ligation, substantial collagen deposition with loss of pancreatic exocrine and endocrine tissue was observed in WT but not in Piezo1^GFAP^-KO mice (Figure 1, H–K). These findings indicate that pressure induced by duct ligation induces pancreatic fibrosis by activating stellate cells independently of acinar cell–mediated pancreatitis.

### Piezo1 causes sustained [Ca^2+^]_i_ elevation in PSCs.

**Figure 2:**
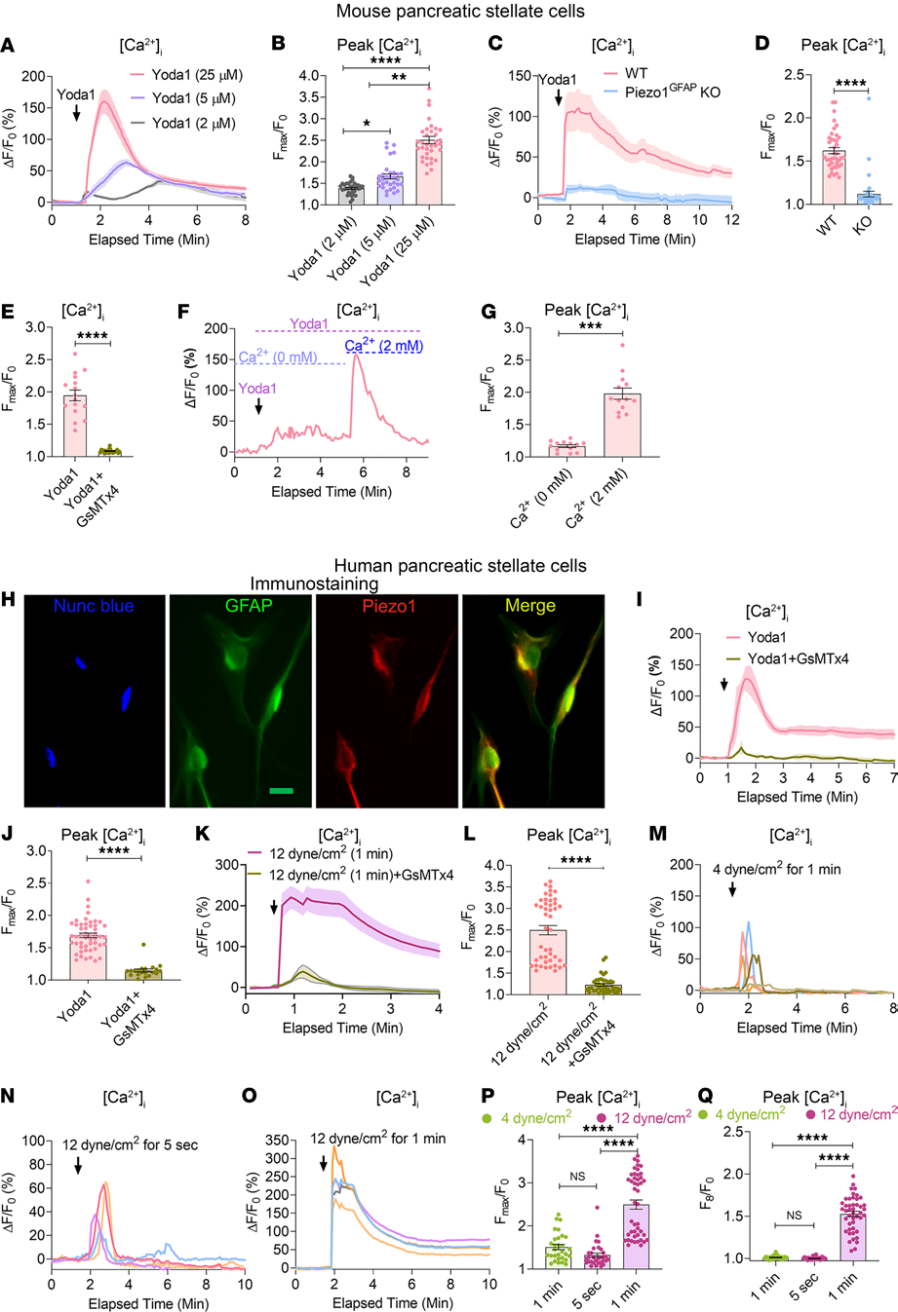
Functional expression of Piezo1 in pancreatic stellate cells. (**A** and **B**) Tracing and graph illustrate the dose-dependent effects of the Piezo1 agonist, Yoda1, on [Ca^2+^]_i_ in mouse PSCs (from 33 to 35 cells). The arrow represents the time of Yoda1 application. (**C** and **D**) Effects of Yoda1 (25 μM) on [Ca^2+^]_i_ are shown in PSCs from WT and Piezo1^GFAP^-KO mice (from 40 cells). (**E**) Effects of Yoda1 on the rise in [Ca^2+^]_i_ was blocked by the Piezo1 antagonist GsMTx4 (5 μM) (from 16 to 21 cells). (**F** and **G**) Yoda1 increased [Ca^2+^]_i_ in the presence and absence of external calcium (from 13 cells). (**H**) Immunostaining of GFAP, nuclei (with Nunc blue), and Piezo1 in human PSCs. Merged images of GFAP (green) and Piezo1 (red) appear as yellow. (**I** and **J**) Yoda1-mediated [Ca^2+^]_i_ rise in human PSCs was blocked with GsMTx4 (5 μM) (from 20 to 50 cells). (**K** and **L**) Tracing and graph represent the effects of fluid shear stress (12 dyne/cm^2^) applied for 1 minute on [Ca^2+^]_i_ with or without GsMTx4 (5 μM) (from 41 to 45 cells). (**M**–**O**) Representative tracings show the effects of fluid shear stress applied at 4 dyne/cm^2^ for 1 minute, 12 dyne/cm^2^ for 5 seconds, and 12 dyne/cm^2^ for 1 minute on [Ca^2+^]_i_ in human PSCs. (**P** and **Q**) Statistical comparisons of the peak and sustained [Ca^2+^]_i_ rise for the fluid shear stresses shown in **M**–**O** (from 30 to 45 cells). The sustained elevation in [Ca^2+^]_i_ was measured at 8 minutes of imaging. Data are presented as mean ± SEM. Statistical analyses were calculated using 2-tailed Student’s *t* test for 2 groups, and multiple groups were analyzed by 1-way ANOVA. *P ≤ 0.05; **P ≤ 0.01, ***P ≤ 0.001; ****P ≤ 0.0001. Scale bar: 10 μm.

It is well known that PSCs produce the excessive ECM proteins in pancreatic fibrosis, so we hypothesized that increased intrapancreatic pressure stimulates increased PSC ECM production. To investigate this possibility, we first cultured mouse PSCs on a Matrigel-coated plate to maintain a quiescent phenotype. As evidence of quiescence, nearly 97% of PSCs retained Bodipy^+^ perinuclear fat droplets (Supplemental Figure 2C). To determine possible mechanoreceptor expression, Piezo1 was identified in PSCs by immunostaining (Figure 1C and Supplemental Figure 2, A and B), and functional activation was demonstrated by dose-dependent increases in [Ca^2+^]_i_ elevation in response to the Piezo1, agonist Yoda1 (11, 22) (Figure 2, A and B). The Yoda1-stimulated increase in [Ca^2+^]_i_ was absent in Piezo1-deleted PSCs (Figure 2, C and D), confirming that the effects of Yoda1 are specific for Piezo1. GsMTx4, a Piezo1 antagonist, blocked the calcium rise produced by Yoda1 (Figure 2E) (23). Removing external calcium abolished the Yoda1-induced calcium influx, and replacing calcium (2 mM) restored calcium entry, proving that the Piezo1-mediated increase in [Ca^2+^]_i_ in PSCs was dependent on external calcium (Figure 2, F and G). High-dose Yoda1 (25 μM) did not affect PSC viability and membrane integrity. All cells responded to the calcium ionophore, ionomycin, after Yoda1 (25 μM) treatment (Supplemental Figure 3). Like mouse PSCs, Piezo1 channels are expressed in human PSCs and are sensitive to GsMTx4 blockade following Yoda1 (25 μM) stimulation (Figure 2, H–J). Mechanical forces such as shear stress and pressure are physiological activators of Piezo1 (13, 16). We applied mechanical force by touching PSCs with a glass pipette. Pressure applied for 1 second produced only a transient rise in [Ca^2+^]_i_ (Supplemental Figure 4). To apply mechanical force for longer periods of time and to avoid cell accommodations to prolonged mechanical pushing, we used fluid shear stress. Prolonged high fluid shear stress is an approach similar to high–fluid pressure situations during obstructive pancreatitis (21, 24, 25). To determine the effects of shear stress on human PSCs, we applied fluid shear stress at 12 dyne/cm^2^ for 1 minute, which produced a sustained increase in [Ca^2+^]_i_ similar to that of Yoda1 (25 μM). These changes were blocked by GsMTx4 (Figure 2, K and L). In contrast to the effects of high shear force, low shear stress (4 dyne/cm^2^ for 1 minute) or high shear stress of shorter duration (12 dyne/cm^2^ for 5 seconds) produced only a transient rise in [Ca^2+^]_i_ (Figure 2, M–Q). These findings indicate that the effects of mechanical force on [Ca^2+^]_i_ are dependent on force and time.

### Piezo1 triggers PSC activation.

**Figure 3:**
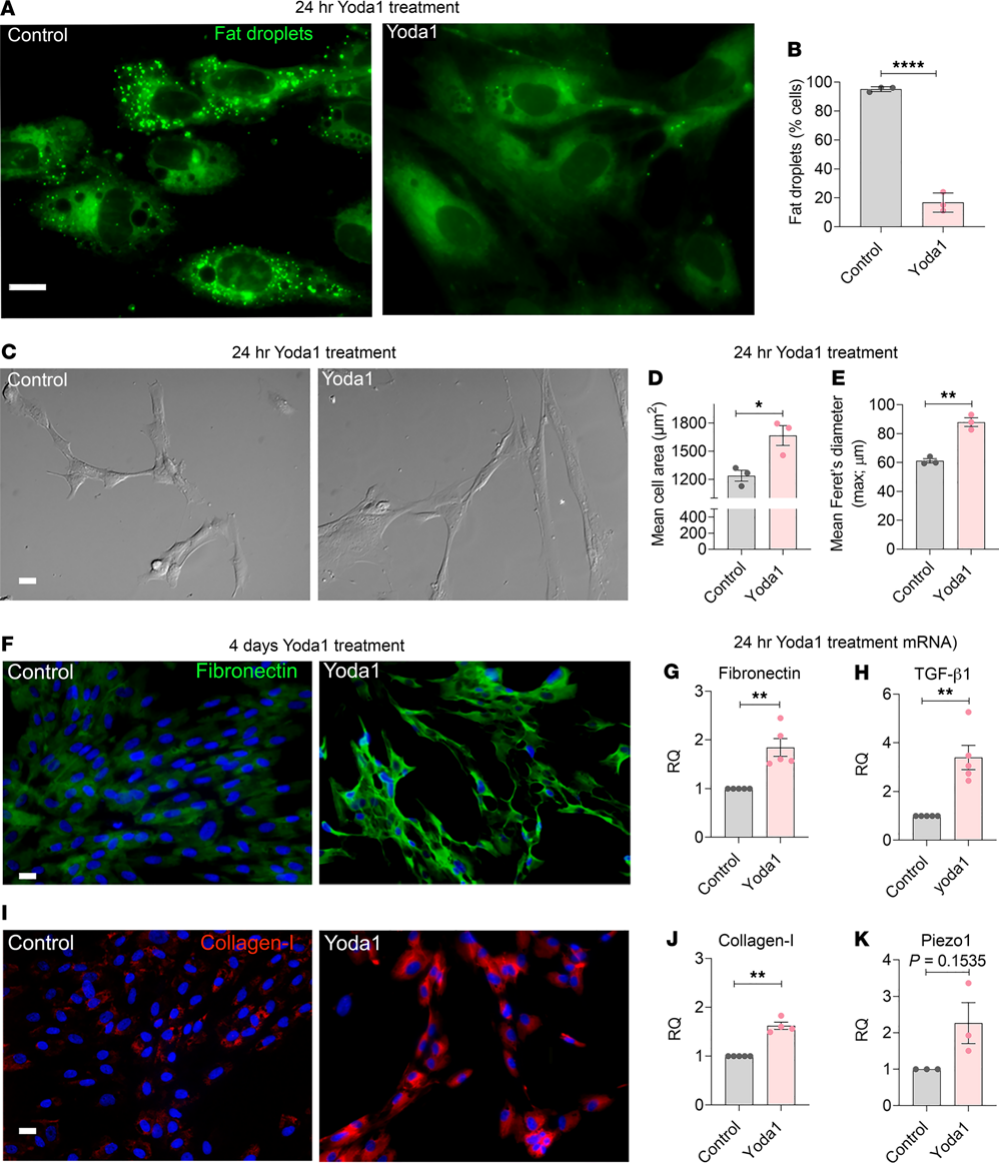
Piezo1 agonist, Yoda1, induces stellate cell activation and fibrosis in vitro. (**A**) Fluorescent dye, Bodipy 493/503 stained fat droplets (green) are visible in human PSCs 24 hours after Yoda1 (25 μM). (**B**) Graph represents the Yoda1-induced loss of fat droplets (from 3 experiments; > 100 cells analyzed for each experiment). (**C**) Differential interference contrast (DIC) images of human PSCs are shown with or without Yoda1 treatment (25 μM for 24 hr). (**D** and **E**) The mean cell area and mean Feret’s diameter (max) of human PSCs with and without Yoda1 (25 μM) (from 3 experiments; > 20 cells analyzed in each experiment). (**F** and **I**) Representative images of fibronectin and collagen type I immunostaining in human PSCs 4 days after Yoda1 (25 μM) (from 3 experiments). (**G**, **H**, **J**, and **K**) mRNA levels of fibronectin, collagen type I, TGF-β1, and Piezo1 in human PSCs 24 hours after treatment with Yoda1 (25 μM) (3–5 experiments). Data are shown as mean ± SEM. Scale bars: 10 μm. Statistical analyses were calculated using 2-tailed Student’s *t* test for 2 groups, and multiple groups were analyzed by 1-way ANOVA. *P ≤ 0.05; **P ≤ 0.01, ****P ≤ 0.0001.

**Figure 4:**
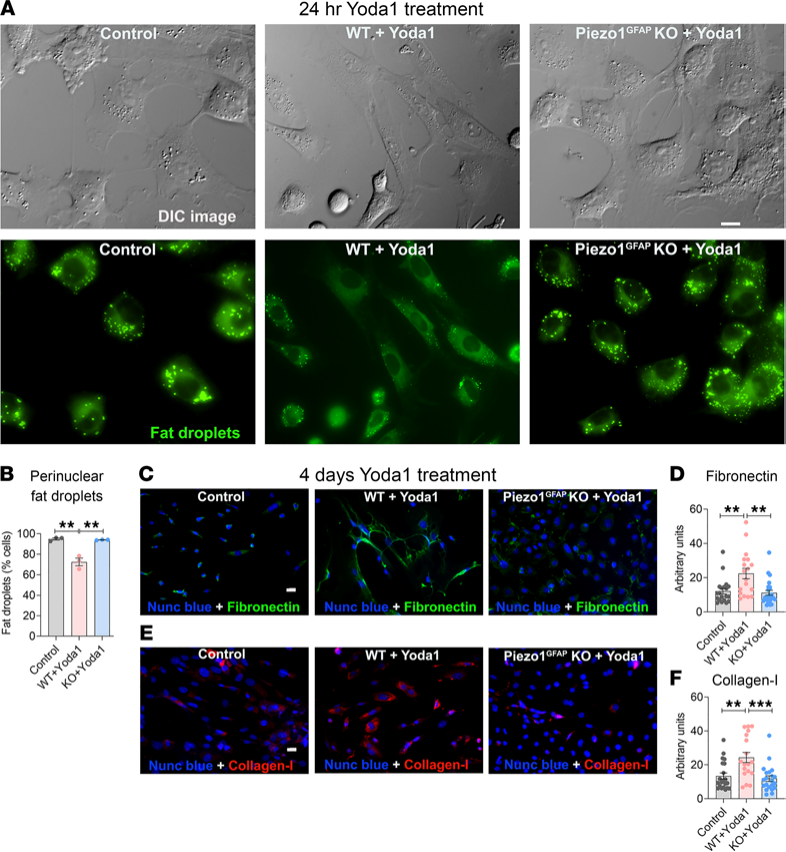
Yoda1 caused stellate cell activation and conversion to a fibroblast phenotype. (**A**) DIC images and Bodipy 493/503–stained fat droplets (green) in WT and Piezo1^GFAP^-KO mouse PSCs without Yoda1 and 24 hours after Yoda1 (25 μM). The images were captured using MetaMorph 7.10.1 software from Molecular Devices. (**B**) Yoda1-induced loss of fat droplets (from 3 experiments; > 100 cells per experiment). (**C** and **E**) Representative images of fibronectin and collagen type I immunostaining in mouse PSCs 4 days after Yoda1 (25 μM) (representative of 3 experiments). (**D** and **F**) Quantification of the fibronectin and collagen type I intensity (arbitrary units) were calculated from data shown in **C** and **E**. Data are shown as mean ± SEM. Scale bars: 10 μm. Statistical analyses were calculated for multiple groups by 1-way ANOVA. **P ≤ 0.01, ***P ≤ 0.001.

In healthy pancreas, quiescent PSCs are characterized by perinuclear, vitamin A–containing fat droplets and low-level expression of ECM proteins such as fibronectin and collagen (17, 26–28). Upon activation, PSCs lose perinuclear fat droplets and produce excess ECM proteins. To determine if Piezo1 has the ability to convert quiescent PSCs to an activated phenotype, we applied Yoda1 (25 μM) to human PSCs. Within 24 hours, nearly 80% of PSCs had lost their perinuclear fat droplets and became elongated with a significant increase in mean cell area and maximum diameter (mean Feret’s diameter-max; Figure 3, A–E) (29, 30). Fibronectin, collagen type I, and Piezo1 mRNA levels were also significantly elevated (Figure 3, G, H, J, and K). After 4 days of Yoda1 treatment, fibronectin and collagen type I immunostaining were abundant (Figure 3, F and I). Like human PSCs, Yoda1 converted mouse PSCs to an activated phenotype with reduced perinuclear fat droplets, changes in cell shape, and increased fibronectin and collagen type I (Figure 4). All the effects of Yoda1 on fibrosis were prevented in Piezo1-deleted PSCs (Figure 4), confirming their Piezo1 dependence.

### High shear stress induces PSC activation and fibrogenic responses in vitro.

**Figure 5:**
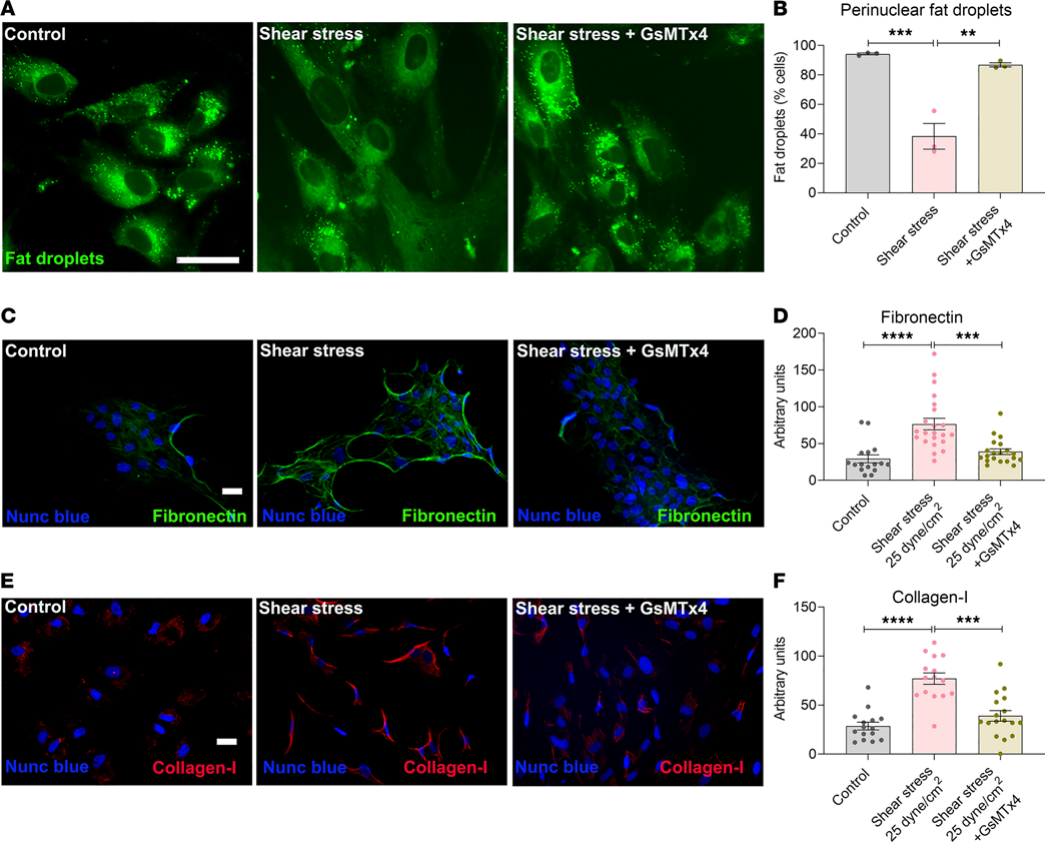
Fluid shear stress–induced stellate cell activation and fibrosis in vitro. (**A**) Fluid shear stress (12 dyne/cm^2^ for 10 minutes) induced loss of fat droplets (green) stained with Bodipy 493/503 in human PSCs with and without GsMTx4 (5 μM). Images were taken 24 hours after shear stress. (**B**) Quantification of reduction in fat droplets 24 hours after shear stress (from 3 experiments and > 50 cells per experiment). (**C** and **E**) Representative images of fibronectin and collagen type I immunostaining in human PSCs 3 days after last application of shear stress. Shear stress (25 dyne/cm^2^ for 10 minutes) was applied twice at an interval of 24 hours. (**D** and **F**) Quantification of the fibronectin and collagen type I intensity was calculated from data shown in **C** and **E**. Data represent the mean ± SEM. Statistical analyses were calculated using 1-way ANOVA. **P ≤ 0.01, ***P ≤ 0.001; ****P ≤ 0.0001. Scale bar: 10 μm.

Application of high shear stress (12 dyne/cm^2^) for 1 minute produced sustained [Ca^2+^]_i_ elevation in PSCs. To determine if physical force mediates stellate cell activation through Piezo1, we studied the effect of high fluid shear stress with and without the Piezo1 blocker, GsMTx4. Shear stress (12 dyne/cm^2^ for 10 minutes) applied to human PSCs significantly reduced the number of perinuclear fat droplets (Figure 5, A and B). Repeated injury to the pancreas, associated with edema and increased pancreatic pressure, leads to chronic pancreatitis (4, 5, 7, 31). Our observation that Yoda1 increased Piezo1 expression (Figure 3K) raised the possibility that repeated exposure to mechanical force may induce a Piezo1-mediated fibrogenic response. To test this hypothesis, we applied shear stress (25 dyne/cm^2^ for 10 minutes) twice at an interval of 24 hours and examined the stellate cell activation and fibrogenic responses in vitro. Importantly, repeated shear stress increased fibronectin and collagen type I in human PSCs (Figure 5, C–F). Treatment with the Piezo1 blocker, GsMTx4, attenuated the shear stress–mediated pathological changes (Figure 5), demonstrating that mechanical force induced stellate cell activation and increased ECM protein synthesis through Piezo1.

### Piezo1 signaling mediates TRPV4 channel opening in PSCs.

**Figure 6:**
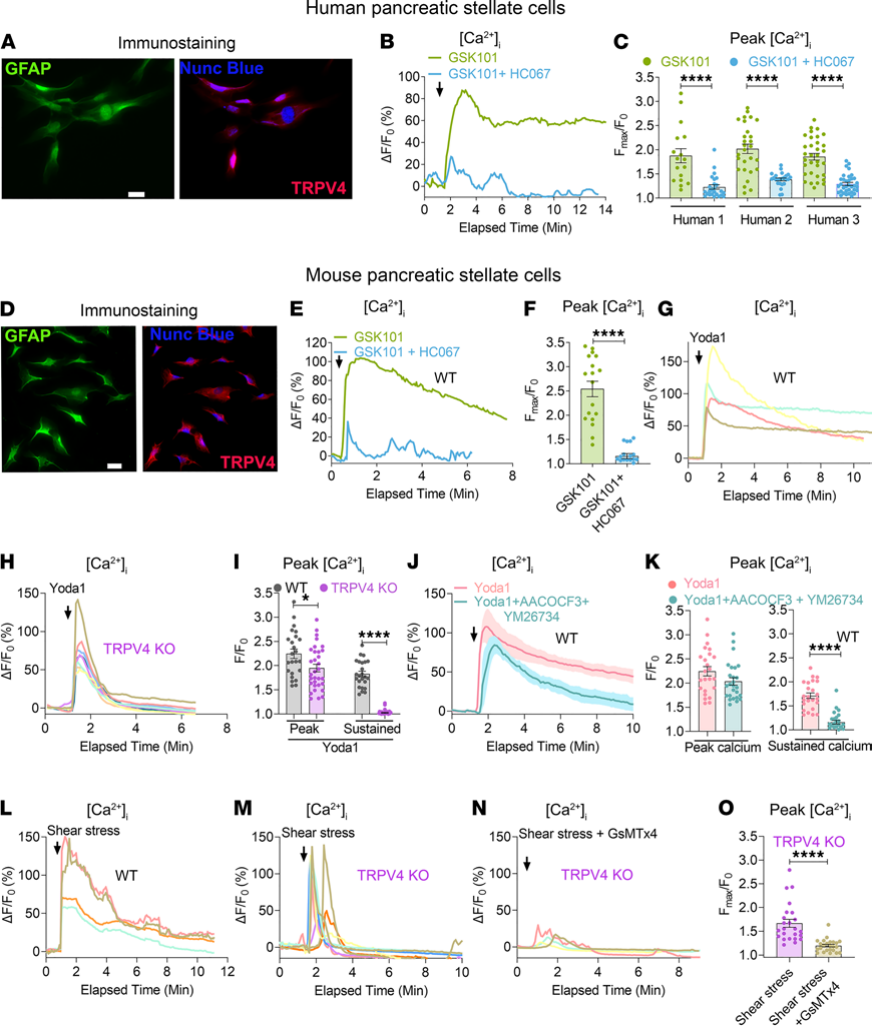
Piezo1 mediates TRPV4 channel opening in pancreatic stellate cells. (**A**) Immunostaining of GFAP and TRPV4 in human PSCs. (**B**) TRPV4 agonist GSK101 (100 nM) effects on [Ca^2+^]_i_ in human PSCs with and without the TRPV4 blocker HC067 (1 μM). (**C**) GSK101 (100 nM) effects on [Ca^2+^]_i_ in PSCs from 3 biological samples were blocked with the TRPV4 antagonist HC067 (1 μM) (from 18 to 37 cells). (**D**) Immunostaining of GFAP and TRPV4 in mouse PSCs. (**E** and **F**) Traces and graph represent the effects of the TRPV4 agonist GSK101 (100 nM) on [Ca^2+^]_i_ in mouse PSCs with and without the TRPV4 blocker HC067 (1 μM) (from 18 cells). (**G**–**I**) Effects of Yoda1 (25 μM) on [Ca^2+^]_i_ in PSCs from WT and TRPV4-KO mice. (**I**) Statistical analyses of peak and sustained [Ca^2+^]_i_ elevation (from 24 to 32 cells). The sustained [Ca^2+^]_i_ elevation was measured at 6 minutes after Yoda1. (**J** and **K**) Effects of phospholipase A2 blockers AACOCF3 (30 μM) and YM26734 (10 μM) on Yoda1-induced (25 μM) [Ca^2+^]_i_ in PSCs (from 24 to 26 cells). The sustained calcium rise was measured at 8 minutes after Yoda1 application. (**L**–**N**) Fluid shear stress (12 dyne/cm^2^) was applied for 1 minute in PSCs from WT and TRPV4-KO mice and TRPV4-KO mice with GsMTx4 (5 μM). In panels **G**, **H**, and **L**–**N**, each colored line represents the response of a single cell. (**O**) Quantification of peak [Ca^2+^]_i_ following shear stress (12 dyne/cm^2^) for 1 minute in TRPV4-KO PSCs with and without GsMTx4 (from 24 cells). Statistical analyses were calculated using 2-tailed Student’s *t* test. *P ≤ 0.05 and ****P ≤ 0.0001. Scale bar: 10 μm.

Recently, we discovered that Piezo1 downstream signaling activates the TRPV4 channel in pancreatic acinar cells and human umbilical vein endothelial cells (HUVECs) (13, 24). Here we detected functional TRPV4 channels in mouse and human PSCs (Figure 6, A–F). The TRPV4 agonist, GSK1016790A, produced a sustained elevation in [Ca^2+^]_i_ in quiescent mouse and human PSCs that was blocked with the TRPV4 blocker HC067047 (Figure 6, B, C, E, and F) (13). Although, under certain circumstances, TRPV4-expressing cells respond to mechanical force, this appears to be an indirect effect, since it has not been demonstrated that mechanical manipulations directly cause TRPV4 channel opening (13, 14, 24, 32). To determine if TRPV4 is involved in Piezo1-mediated changes in [Ca^2+^]_i_ in PSCs, we isolated cells from TRPV4-KO mice. Yoda1 (25 μM) or prolonged, high shear stress (12 dyne/cm^2^ for 1 minute), which normally produce sustained elevations in [Ca^2+^]_i_, caused only transient calcium elevations in TRPV4-null PSCs (Figure 6, G–I, L, and M). 5′,6′-Epoxyeicosatrienoic acid is an endogenous activator of the TRPV4 channel produced from arachidonic acid via a PLA2-AA cytochrome P450 epoxygenase–dependent pathway (32, 33). To determine if Piezo1 activates PLA2-AA, we used secretory and cytosolic PLA2 inhibitors YM26734 and AACOCF3 (13). Together, YM26734 and AACOCF3 significantly inhibited the Yoda1-mediated sustained elevation in [Ca^2+^]_i_ in PSCs from WT mice (Figure 6, J and K). Additionally, GsMTx4, blocked the effects of high shear stress (12 dyne/cm^2^ for 1 minute) on the transient [Ca^2+^]_i_ elevation in TRPV4-null PSCs, confirming that the initial transient calcium influx was due to Piezo1, while subsequent TRPV4 channel opening was responsible for the sustained rise in [Ca^2+^]_i_ (Figure 6, N and O).

### TRPV4-KO mice were protected from pressure-induced pancreatic fibrosis.

**Figure 7:**
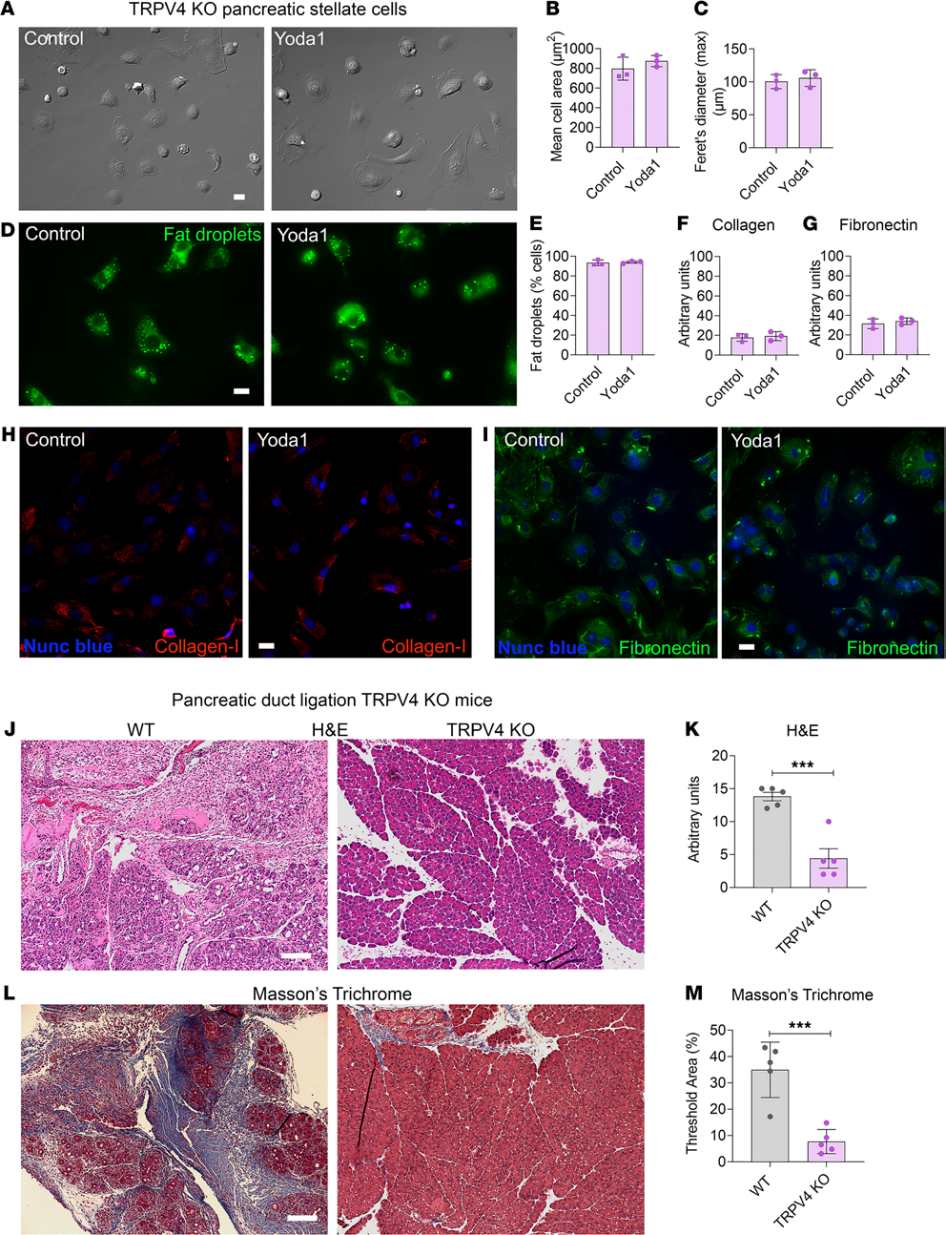
TRPV4-KO mice were protected from pancreatic duct ligation–induced fibrosis. (**A** and **D**) DIC and Bodipy 493/503–stained images of PSCs from TRPV4-KO mice 24 hours after Yoda1 (25 μM). (**B** and **C**) Mean cell area and Feret’s diameter (max) of PSCs 24 hours after Yoda1 (25 μM) (from 3 experiments with 20 cells each). (**E**) Loss of fat droplets in PSCs following Yoda1 (25 μM) (from 3 experiments and > 100 cells). (**F** and **G**) Quantification of collagen type I and fibronectin immunostaining in PSCs from TRPV4-KO mice 4 days after Yoda1 (25 μM). (**H** and **I**) Representative images of collagen type I and fibronectin staining for the data shown in **F** and **G**. (**J**–**M**) Pancreatic duct ligation (PDL) at the tail region of the pancreas induced chronic pancreatitis and fibrosis in WT and TRPV4-KO mice. Eight days after PDL, chronic pancreatitis and fibrosis parameters of the tail region included (**J**) H&E staining, (**K**) H&E score, (**L**) Masson’s trichrome staining, and (**M**) area of WT and TRPV4-KO mice (*n =* 5). Statistical comparisons were made using 2-tailed Student’s *t* test. ***P ≤ 0.001. Scale bar: 100 μm.

Having determined that the sustained elevation in [Ca^2+^]_i_ produced by Piezo1 activation requires TRPV4 opening, we proposed that TRPV4 was responsible for stellate cell activation. To test this possibility, we treated PSCs isolated from TRPV4-KO mice with Yoda1 (25 μM). In contrast to WT PSCs, Yoda1 did not activate PSCs from TRPV4-KO mice, and no changes in PSC activation parameters (cell shape, perinuclear fat droplet abundance, and ECM protein expression) were observed (Figure 7, A–I). If pathological effects of Piezo1 (high-pressure–induced stellate cell activation and fibrosis) require TRPV4 channels, we would expect that mice lacking TRPV4 would be protected against pressure-induced chronic pancreatitis and fibrosis. As shown in Figure 7, J–M, mice lacking TRPV4 channels were protected from PDL-induced pancreatic fibrosis.

## Discussion

Pancreatic fibrosis is an irreversible complication of chronic pancreatitis that is often accompanied by loss of endocrine and exocrine function (6–8). Fibrosis is composed of ECM produced by activated PSCs, which also secrete proinflammatory cytokines that may amplify pancreatic inflammation and accelerate the loss of acinar and islet cells (34). Pancreatic fibrosis also increases the risk of pancreatic ductal adenocarcinoma (PDAC) (10). The dense desmoplasia found in PDAC is a product of a subtype of activated PSCs known as cancer-associated fibroblasts and poses a major hurdle for chemotherapeutic-based drug delivery. Effective antifibrotic therapies are lacking; therefore, most current efforts are directed at preventing fibrosis by blocking PSC activation. The 2 most common factors leading to chronic pancreatitis are heavy alcohol use and conditions producing sustained elevations in pancreatic duct pressure, such as duct strictures, cysts, pseudocysts, and obstructive tumors. We show here that PSCs exhibit pressure sensitivity by virtue of their expression of the mechanically activated ion channel Piezo1 and that activation of Piezo1 initiates a fibrogenic response. Complete manifestation of the pathological consequences of Piezo1 activation requires calcium-triggered TRPV4 channel opening and its accompanying calcium influx.

The current study demonstrates that increased intraductal pressure causes pancreatic fibrosis mediated by PSCs and that PSC sensitivity to pressure is mediated by Piezo1 activation. Brief high shear stress or low shear stress for longer times produced transient increases in [Ca^2+^]_i_ that were insufficient to activate PSCs. In contrast, Yoda1 or sustained higher shear force produced a sustained elevation in [Ca^2+^]_i_ and induced PSC activation, manifest by cell elongation, loss of perinuclear fat droplets, and stimulation of profibrotic TGF-β1 and ECM protein (e.g., fibronectin and collagen type I) gene expression. Our findings that Piezo1^GFAP^-KO mice were protected from pressure-induced fibrosis suggest that a Piezo1 blocker could be a possible treatment for pancreatic fibrosis.

Pancreatic fibrosis is an active inflammatory process, accompanied by cell-to-cell contact and dynamic production of inflammatory molecules (35). Activated PSCs secrete IL-6, IL-1β, monocyte-specific chemokine (MCP-1), and TNF-α; activate tissue-resident macrophages; and recruit inflammatory monocytes, which are major regulators of fibrosis (35–39). In human fibrotic tissue and a rat model of chronic pancreatitis, macrophages are in close proximity to PSCs (36) and exacerbate the progression of pancreatic fibrosis through the production of TNF-α and TGF-β1 (36). Importantly, mechanical forces generate proinflammatory responses in macrophages and monocytes in a Piezo1-dependent manner (40–43). This is illustrated by the finding that macrophages lacking Piezo1 exhibited decreased inflammation and enhanced wound healing (43). Piezo1 signaling in myeloid cells also exacerbated a mouse model of pulmonary fibrosis (41, 44); thus, it appears that Piezo1 can induce fibrosis by acting both directly on stellate cells and indirectly through inflammatory cells. It is conceivable that a blocker of Piezo1 or its downstream signaling pathways could inhibit stellate cell activation and reduce the fibrogenic responses triggered by inflammatory immune cells.

We recently reported that pancreatic acinar cells express Piezo1 and that elevated pancreatic pressure can cause pancreatitis (11, 24). During the course of pancreatitis, stellate cells can be activated by proinflammatory molecules, some of which are generated by acinar cells (27, 39, 45–47). Thus, it is possible that fibrosis may result from pressure acting directly on PSCs or indirectly on acinar cells through the induction of pancreatitis and subsequent stellate cell activation. In the current study, we observed that PDL produced extensive pancreatic fibrosis in pancreata of WT and Piezo1^aci^-KO mice, but not Piezo1^GFAP^-KO mice, indicating that Piezo1 channels in stellate cells rather than acinar cells are responsible for pressure-induced fibrosis. Although acinar cell–mediated inflammatory signaling undoubtedly contributes to the development of chronic pancreatic and fibrosis under certain conditions (46, 47), it appears that elevated pancreatic pressure directly promotes stellate cell activation and fibrosis.

TGF-β1 is a major profibrogenic cytokine and a target for antifibrotic therapies (48, 49). Regulation of TGF-β1 function depends on site-specific activation by integrins (50–52), and recently, it has been demonstrated that mechanical activation of Piezo1 converts inactive integrins to an active form (50–52). In addition to the role of Piezo1 in activation of TGF-β1, our results demonstrate that Piezo1 increases TGF-β1 at the transcriptional level.

Discovery of TRPV4 in PSCs raised the possibility that the pathological effects of Piezo1 on generation of fibrosis may require TRPV4 (53, 54). Our results indicate that Piezo1 senses mechanical force and initiates calcium signaling, resulting in TRPV4 activation. In the absence of TRPV4, mechanical force or Yoda1 did not generate the sustained elevation in [Ca^2+^]_i_ that was necessary to alter PSC morphology, modify perinuclear fat droplet abundance, or initiate fibrogenic responses. Although under certain circumstances, TRPV4 has been variously reported as mechanosensitive, this has not been demonstrated in cell-free systems (14, 55, 56), and it seems likely that mechanoreceptor properties that were attributed to TRPV4 may be due to true mechanically activated ion channels like Piezo1 that happen to be coexpressed (11, 13, 24). PSCs appear to be another example of Piezo1 and TRPV4 interdependence.

The observation that Yoda1 elevated Piezo1 and TRPV4 mRNA levels in PSCs from WT mice suggests that prolonged pressure may increase the fibrogenic response (Figure 3K and Supplemental Figure 5). It will be interesting to evaluate Piezo1 and TRPV4 in stellate cells of patients with chronic obstructive pancreatitis and to determine if administration of a Piezo1 or TRPV4 blocker can block or reverse obstructive chronic pancreatitis and fibrosis in humans. TRPV4 is expressed in many organs that have mechanosensing properties and exhibit fibrosis in response to injury including the heart, lung, kidney, liver, skin, and intestine (57–59). In the liver, TRPV4 expression was increased in hepatic fibrosis and linked to TGF-β1–induced hepatic stellate cell activation. Like hepatic fibrosis, TRPV4 was upregulated in fibrotic pulmonary tissue and TRPV4-KO mice were protected from pulmonary fibrosis (59). In the heart, TRPV4 converts fibroblasts to a myofibroblast phenotype through a TGF-β1–mediated pathway (57). Thus, TRPV4 is an established mediator of tissue fibrosis. In addition to our findings in the pancreas, TRPV4 expression was found to be upregulated in a model of alcohol- and high-fat diet–induced pancreatitis (53), and TRPV4 is expressed in macrophages and linked with inflammation (60–62). Our observations together with these findings support a possible strategy for preventing or treating pancreatic fibrosis by blocking Piezo1 or TRPV4 channels.

## Methods

### Animals.

*Piezo1^fl/fl^* mice were a gift from A. Patapoutian (Scripps Research; ref. 63). To generate *Piezo1* deletion in stellate cells, *Piezo1^fl/fl^* mice were crossed with *B6.Cg-Tg(GFAP-cre/ERT2)505Fmv/J* mice (The Jackson Laboratory) to generate the mouse line *B6.Cg-Tg(GFAP-cre/ERT2)*; *piezo1^fl/fl^*. To generate conditional genetic *Piezo1* deletion in stellate cells, 40 mg of tamoxifen/kg body weight (MilliporeSigma, T5648) was injected i.p. per day for 5 consecutive days (11). The mice were used 8 days after the last tamoxifen injection. The mouse lines *B6.Cg-Tg (GFAP-cre/ERT2)*; *piezo1^fl/fl^* and *ptf1a^CreERTM^; piezo1^fl/fl^* (generated as described in ref. 11) after tamoxifen injection were referred to as Piezo1^GFAP^-KO and Piezo1^aci^-KO, respectively (11). *Piezo1^fl/fl^* mice were used as WT in the experiments with Piezo1^aci^-KO and Piezo1^GFAP^-KO mice. Seven- to 12-week-old mice (both male and female) were used in the experiments. A small piece of tail of each mouse was used for genotyping. The mouse line with *Trpv4* gene deletion (referred to as TRPV4-KO) was obtained from Wolfgang Liedtke (Department of Neurology, Duke University; ref. 64) and then bred in-house. C57BL/6J mice (The Jackson Laboratory) were used as WT mice in the experiments with TRPV4-KO mice. Mice were housed under standard 12-hour light/12-hour dark conditions.

### In vivo experiments.

PDL of the tail region was performed as previously described (21, 24). The pancreas was visualized using a stereomicroscope, and the tail region of the main pancreatic duct was ligated with 7-0 (0.5 metric) nonabsorbable, Prolene suture without damaging underlying arteries and veins. Mice suffering injury to any underlying blood vessels were excluded from the experiment. Mice were sacrificed at day 8 or day 30 after surgery.

### In vitro experiments.

Mouse and human PSCs were isolated using collagenase digestion (26). Modified Krebs Henseleit Buffer (KHB) solution (100 mL) was prepared as described previously (11). Pancreatic tissue was digested with 2 mg of collagenase NB 8 (SERVA, catalog 17456) dissolved in 10 mL of modified KHB solution containing 1 mg soybean trypsin inhibitor (SBTI 1-S; MilliporeSigma, catalog T9003) and 20 mg BSA (Thermo Fisher Scientific; BP1600-100).Digestion solution (5 mL) was used to inflate the pancreas. Pancreas with 5 mL of digestion solution was incubated in a shaking water bath for 10 minutes at 37°C. The solution was then discarded. The pancreas tissue was cut into small pieces and digested with fresh 5 mL digestion buffer. Tissue was incubated in a shaking water bath for 40 minutes at 37°C. The cells were then separated from tissue by pipetting up-down with a 10 mL pipette and passed sequentially through a 70 μm and a 40 μm cell strainer. The filtrate was centrifuged at 150*g* for 3 minutes at room temperature. The cells were washed with 10 mL Leibovitz’s media (Thermo Fisher Scientific, catalog 11415-064), passed through a 20 μm filter, and centrifuged at 80*g* for 3 minutes at room temperature. The supernate was removed, and isolated stellate cells were plated on a thin-layered Matrigel-coated glass bottom culture plate (MatTek, P35G-0-14-C). Before plating the cells, Matrigel solution (Corning, catalog 354234) at a ratio of 1.5:100 DMEM/F12 (Thermo Fisher Scientific, catalog 11330-032) was poured onto the culture plate and incubated for 2 hours at 37°C to form a thin layer of Matrigel coating. Matrigel mixture was removed, and the plate was washed with PBS before cells were plated. Cell culture media, DMEM/F12 with 5% FBS was used for mouse stellate cells. Fresh, human pancreatic tissue (provided by Duke University’s BioRepository & Precision Pathology Center under IRB approval) was digested with collagenase as described above with modifications. Collagenase (2.5 mg) in 10 mL of modified KHB solution was used for digestion. The digested tissues were filtered through a 100 μm cell strainer. The cells were cultured with DMEM/F12 with 10% FBS in a Matrigel-coated plate. After 24 hours, the cell media was replaced with fresh media to remove unattached and dead cells. After 2 days, cells were immunostained for GFAP and used for experiments. Perinuclear fat droplets were stained with BODIPY 493/503 (4,4-Difluoro-1,3,5,7,8-Pentamethyl-4-Bora-3a,4a-Diaza-*s*-Indacene; Invitrogen, catalog D3922) to confirm stellate cell quiescence. The cell viability following Yoda1 treatment was analyzed using the Live/Dead cell imaging kit (Thermo Fisher Scientific, catalog R37601) (24, 65). RNAs were isolated using the RiboPure Kit (Invitrogen, catalog AM1924) according to manufacturer’s instructions (11).

### Shear stress and mechanical pushing.

Fluid shear stress and mechanical pushing applied to stellate cells were achieved as previously described (15). Parallel-plate fluid flow chambers (μ-Slide I 0.4 Luer, or μ-Slide I 0.2 Luer from Ibidi) were used for fluid shear stress experiments. The fluid shear stress (τ) was calculated using the formula: τ = η × 104.7.6 φ for μ-Slide I 0.4 Luer and τ = η × 330.4 φ for μ-Slide I 0.2 Luer, where η represents viscosity of the medium and φ represents flow rate (according to the manufacturer’s instructions, Ibidi). Mechanical pushing was achieved by applying a blunt borosilicate glass pipette once to the cell membrane with a deflection of 2–3 μm for 1 second using a micromanipulator (World Precision Instruments).

### Immunostaining.

Mouse and human PSCs were washed with PBS (pH 7.4) and fixed with 4% paraformaldehyde for 10 minutes at room temperature. The fixed cells were treated with 0.1% Triton X-100 (13). Cells were immunostained with a rabbit anti–TRPV4 antiserum (Alomone, ACC-034, 1:250; ref. 66), rabbit anti–Piezo1 antiserum (Alomone, APC-087, 1:300; ref. 11), rabbit anti-fibronectin antibody (Abcam, ab2413), rabbit anti–collagen type I antibody (Abcam, ab34710), or chicken anti-GFAP antibody (Abcam, 4674) for mouse PSCs or rabbit anti-GFAP (Cell Signaling Technology,12389) for human PSCs overnight at 2°C–8°C. Secondary antibodies included DyLight 488–conjugated anti–chicken IgG (Jackson ImmunoResearch, 703-546-155), DyLight 488–conjugated anti–rabbit IgG (Jackson ImmunoResearch, 711-546-152), or CY 3-conjugated anti–mouse IgG (Jackson ImmunoResearch, 715-166-150), used for 1 hour at room temperature. Nuclei were stained with Nunc blue (Invitrogen, R37606). All staining images were taken with a Zeiss Axio observer Z1 with a 20× or 40× objective.

### Ca^2+^ imaging.

Calcium 6-QF (Molecular Devices) dye was used for live-cell calcium imaging as described previously (11). Cells were imaged in HBSS buffer with 2 mM Ca^2+^. Faintly and highly fluorescent loaded cells were excluded from analysis. The chemicals used in calcium imaging experiments included: GsMTx4 (Abcam, catalog ab141871), Yoda1 (Tocris, catalog 5586), GSK1016790A (MilliporeSigma, catalog G0798), HC067047 (Tocris, catalog 4100), YM26734 (Tocris, catalog 2522), and AACOCF3 (Tocris, catalog 1462).

### Histologic grading.

Head and tail regions of the pancreas were embedded in paraffin and sectioned at 5 μm thick. The tissue sections were stained with Masson’s trichrome or H&E. The images were captured on an EVOS microscope using an Olympus PlanApo 10× objective. The histological scores for chronic pancreatitis severity were calculated as previously described (67). The severity of chronic pancreatitis was graded by considering 5 pathological parameters (inflammatory infiltrate, atrophy, intralobular fibrosis, perilobular fibrosis, and interlobular fibrosis), and each category was scored on a scale of 0 (no injury) to 3 (maximum injury). Total scoring was presented after adding all pathologic parameter scores and was on a scale of 0 (no injury) to 15 (maximum injury). The degree of fibrosis was measured by quantifying the areas stained with Masson’s trichrome (68).

### Statistics.

Data were analyzed using GraphPad Prism 9. Results were represented as mean ± SEM. Two-tailed Student’s *t* test was used for 2-group comparisons, and 1-way ANOVA with Tukey’s multiple-comparison test was used for multigroup data sets. *P* < 0.05 was considered significant. *P ≤ 0.05; **P ≤ 0.01; ***P ≤ 0.001; ****P ≤ 0.0001.

### Study approval.

Experimental protocols and all studies were performed with approval from the IACUC and IRB of Duke University (protocol number Pro00035974).

## Author contributions

SMS designed and performed the experiments and wrote the manuscript. JMJR designed and performed experiments and helped write the manuscript. SRV provided technical advice and helped write the manuscript. RAL helped design experiments, helped write the manuscript, and provided funding for the work. All authors reviewed and approved the manuscript.

## Supplementary Material

Supplemental data

## Version 1 04/22/2022

Electronic publication
