# Supplementary material for: Piezo1-mediated stellate cell activation causes pressure-induced pancreatic fibrosis in mice
Source: JCI Insight. 2022 Apr 22;7(8):e158288. doi: 10.1172/jci.insight.158288 (PMC9089793; doi:10.1172/jci.insight.158288)
Supplement: Supplemental data [file jciinsight-7-158288-s039.pdf]

Supplemental Materials

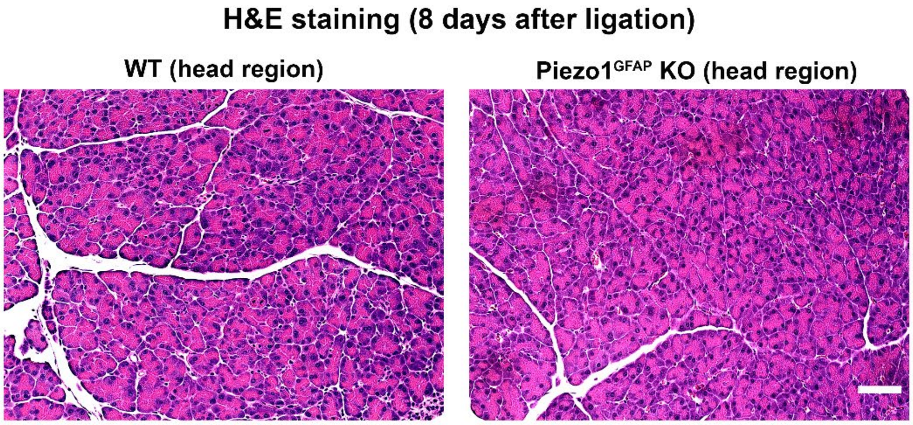

Supplemental Figure 1. Pancreatic duct ligation at tail region did not affect the head of the pancreas. Representative H&E stained images of the pancreatic head region of wildtype (WT) and Piezo1^{GFAP} KO mice 8 days after PDL (n = 3-7).

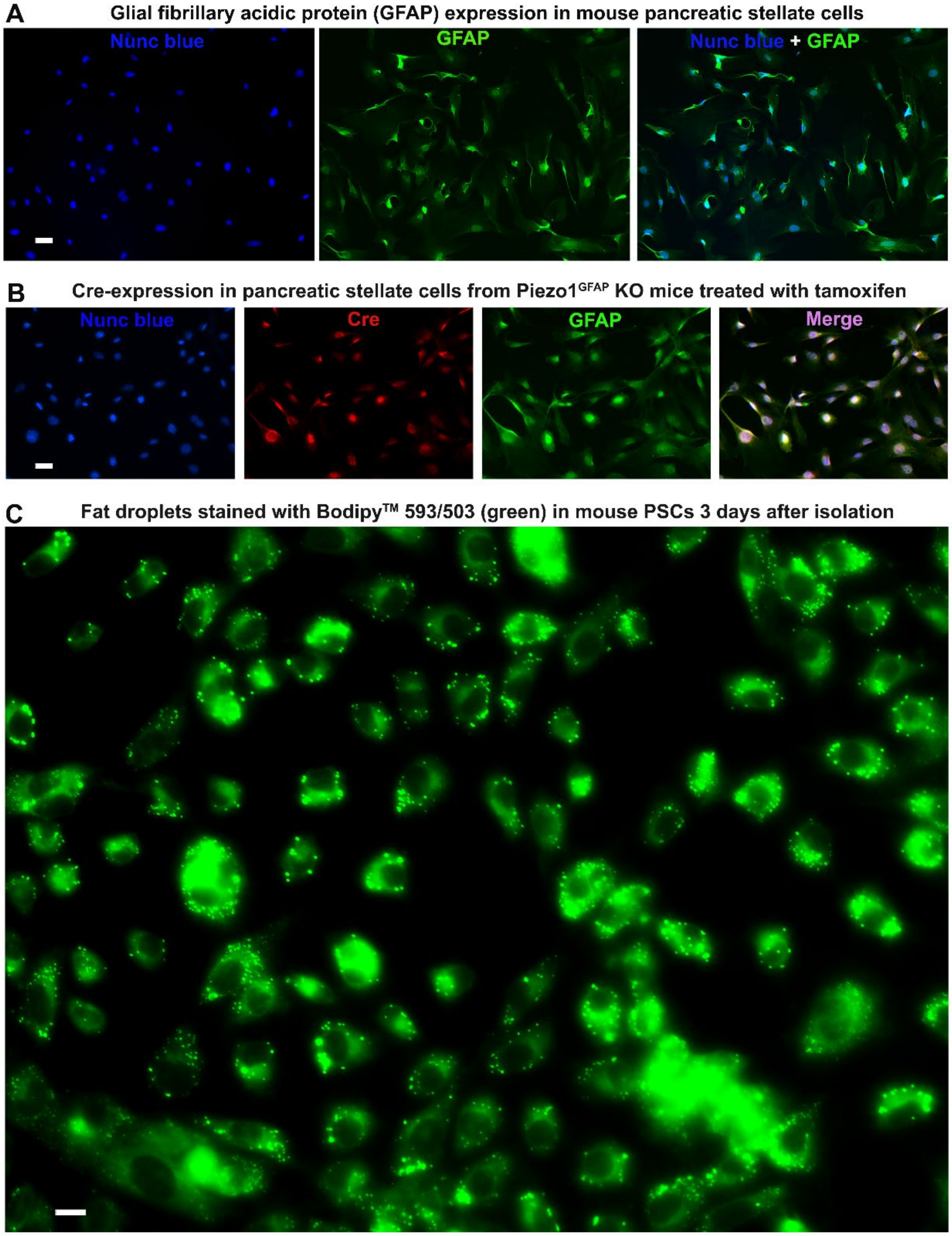

Supplemental Figure 2. Piezo1 deletion in GFAP-expressing PSCs. (A) Images showing expression of GFAP (green) in mouse PSCs after 3 days in culture. Cell nuclei were stained with Nunc blue. (B) PSCs expressing Cre protein from the mouse line B6.Cg-Tg (GFAP-cre/ERT2); *Piezo1*$^{fl/fl}$ after tamoxifen injection (referred to as Piezo1^{GFAP} KO mice) confirm deletion of Piezo1 in PSCs. Scale bar: 20 µm. All cells (Nunc blue), expressing Cre protein, and GFAP appear as merged images. (C) Quiescent PSCs containing perinuclear fat droplets (stained with Bodipy^{TM} 593/503) were cultured on a Matrigel coated plate. Scale bar: 10 µm.

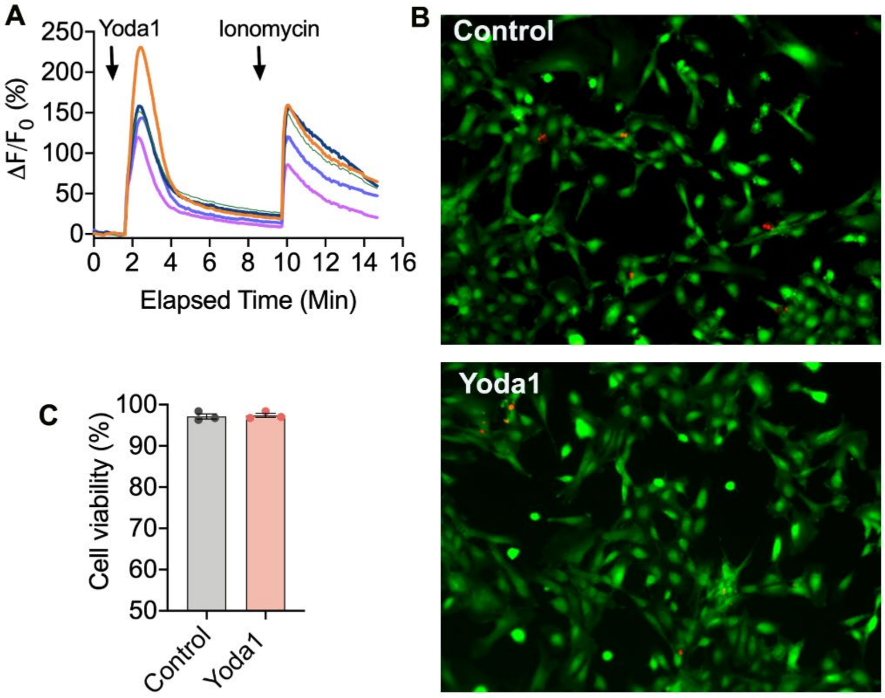

Supplemental Figure 3. High dose Yoda1 did not affect PSC viability and membrane integrity. (A) Mouse PSCs responded to ionomycin (1 µM) after Yoda1 (25 µM) treatment. (B and C) PSCs were treated with Yoda1(25 µM) for 2 hrs afterwhich the viability of stellate cells was analyzed using the Live/Dead Cell Imaging Kit (Thermo Fisher Scientific, Catalog # R37601). Representative images show live (green) and dead (red) cells. (C) The graph represents viable cells with and without Yoda1 treatment (n=3 experiments).

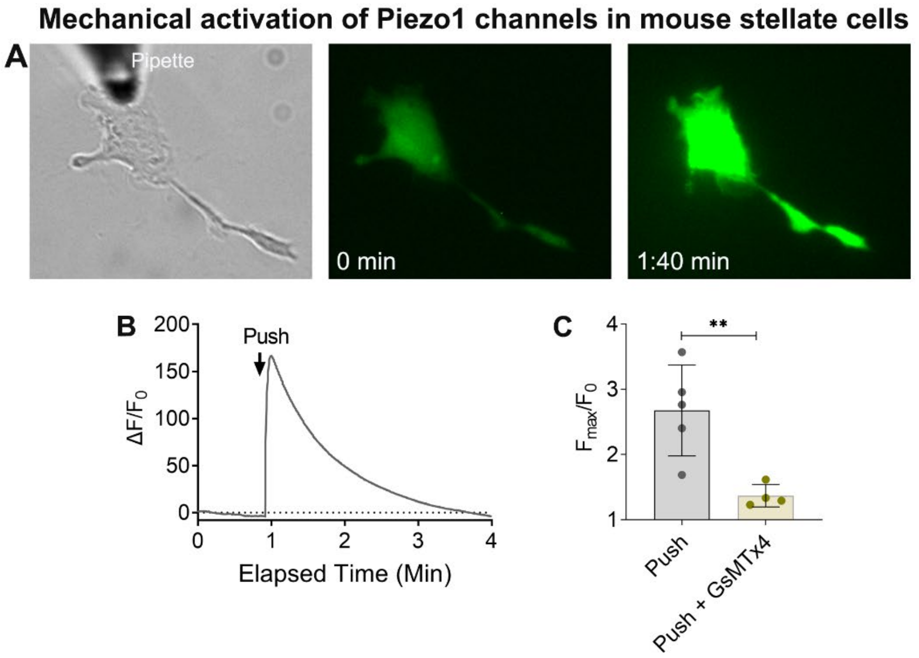

Supplemental Figure 4. Mechanical pushing increases [Ca^{2+}]$_i$ in PSCs. (A) Bright field and live-cell images of mouse PSC at time 0 and at 1:40 (min:s) after mechanical pushing with a blunt tip glass pipette for 1 sec. (B) Representative [Ca^{2+}]$_i$ profile from a single mouse PSC during the course of mechanical pushing. (C) Graph showing peak [Ca^{2+}]$_i$ levels following mechanical pushing in PSCs with and without GsMTx4 (2.5 µM). **P<0.01, n=4-5.

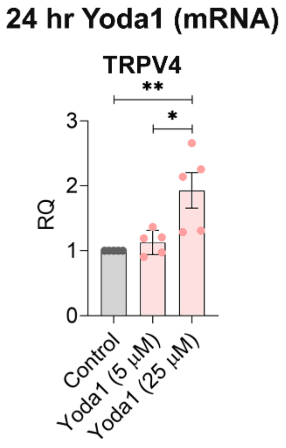

Supplemental Figure 5. Piezo1 agonist, Yoda1, upregulates TRPV4 expression in PSCs. mRNA levels of TRPV4 in human PSCs 24 hrs after treatment with Yoda1 (5 µm and 25 µM). *P<0.05, **P<0.01, n=5.
